# Functionality of Embryo Sacs in Pear Cultivars ‘Ingeborg’ and ‘Celina’ as Related to Fruit Set under Nordic Climate

**DOI:** 10.3390/plants9121716

**Published:** 2020-12-05

**Authors:** Radosav Cerović, Milica Fotirić Akšić, Milena Đorđević, Mekjell Meland

**Affiliations:** 1 Innovation Centre at Faculty of Technology and Metallurgy, University of Belgrade, Karnegijeva 4, 11120 Belgrade, Serbia; radosav.cerovic@gmail.com; 2 Faculty of Agriculture, University of Belgrade, Nemanjina 6, 11080 Belgrade, Serbia; fotiric@agrif.bg.ac.rs; 3 Fruit Research Institute, Čačak, Kralja Petra I/9, 32000 Čačak, Serbia; mdjordjevic@institut-cacak.org; 4 Norwegian Institute of Bioeconomy Research, NIBIO Ullensvang, N-5781 Lofthus, Norway

**Keywords:** *Pyrus communis* L., cultivars, embryo sac, viability, fertilization success, fruit set

## Abstract

Since the European pear (*Pyrus communis* L.) is a self-incompatible fruit species, synchrony and compatibility between female parts of the mother plant and male gametes from the pollen donor must be fulfilled. Besides pollination and fertilization, normal embryo and zygote development is one of the prerequisites for the satisfactory yields in pears. The main goal of this experiment was to investigate the functionality of embryo sacs and the embryo’s early stages of growth in relation to the fruit set of diploid (‘Celina’) and the triploid (‘Ingeborg’) pear cultivars under specific Norwegian climatic conditions. For this purpose, flowers were collected at the beginning of flowering, and on the third, sixth, ninth, and twelfth days after the beginning of this phenophase for two consecutive years. Ovaries were dehydrated, embedded in paraffin wax, sectioned, stained, and observed under the light microscope. In the analyzed cultivars, results showed different tendencies in embryo sac development and degradation processes, in both experimental years, which is probably due to the genetic background of the examined cultivars. Also, fertilization success and fruit set were higher in the second year of study due to the higher average temperature during the flowering period. Diploid cultivar ‘Celina’ showed much better adaptation to high temperatures in relation to triploid cultivar ‘Ingeborg’.

## 1. Introduction

The European pear (*Pyrus communis* L.) is a self-incompatible fruit species (although several studies reported self-compatibility in certain pear cultivars), which is an important reproductive barrier influencing yields. For this reason, overcoming self-incompatibility is one of the most important aims of pear breeding. 

The pear cultivars ‘Ingeborg’ (3n), which is obtained from crossing between ‘Conference’ unreduced egg cell and ‘Bonne Louise’ pollen, and ‘Celina/QTee^®^’ (‘Colorée de Juillet’ × ‘Williams’) are the main commercial pear cultivars grown in Norway [1,2,3]. In addition, significant ‘Celina’ areas are planted in mainly the countries Belgium, The Netherlands, and South Africa as a club cultivar [4]. Both cultivars are developed from the Norwegian breeding program Graminor Ltd. (Hamar, Norway) and have good pomological traits. However, fruit set and yields can be rather low compared to the industry standard in Norway. Depending on the year, unfavorable environmental conditions during flowering can have a very negative effect on fruit set and yield quantity in Norwegian pear orchards. Therefore, it is important to secure good pollination by planting suitable pollinizer genotypes together with the main cultivars [5,6]. 

Gamete development, pollen competition and selection, and synchrony in male–female functionality during mating and in postzygotic stages are important reproductive phases, due to which plants need to be adapted to different, and sometimes unfavorable, environmental conditions [7]. Both pistil and anthers are under the strong influence of changing temperatures during the flower development, before the flowering and post flowering time [8]. Due to this, a mathematical modeling of pollen tubes growth through the pistil and the duration of ovule viability has been developed for some fruit species [9]. Temperature can influence on different phases of reproductive processes, such as stigmatic receptivity [10], pollen germination and pollen tube growth [11,12,13], ovule viability [14,15], and fruit set [16,17]. 

Ovule formation, macrosporogenesis, and macrogametogenesis (the development of embryo sac) are closely connected processes in pear flower development [18,19]. Pollination, pollen tube elongation from stigma to ovule and its entry to the embryo sac are key moments following possible appearance signals for reorganization of the egg apparatus and other female parts [20]. A normal embryo sac and embryo progression are prerequisite to get satisfactory yield [21]. Also, in some cases, the viability of the embryo sacs can be short, following the appearance of early degeneration that limits fertilization success [22]. Nutritional and abiotic stresses during early female development can lead to embryo abortion [23].

On the other hand, unbalanced and sterile gametes with unusual chromosome numbers (aneuploidy) are in most cases originated from triploids. This leads to high number of copies of a certain gene present in a genome, from additive to dominant gene expression of a single allele, and unequal expression of maternal and paternal genes [24]. However, triploids can have normally formed seeds, including apple (triploid × diploid or diploid × triploid) [25], grape [26], and blueberry [27]. In some triploid pear accessions, female fertility can be reduced and differs between genotypes [28]. The egg apparatus of triploid apple cultivars matures 2–3 days after flower opens, unlike diploid cultivars where the egg cell together with synergids are completely mature just after the flower opening, and ready to accept pollen tube and sperm cells [29]. 

Synchrony and regularity of the embryo sac development directly affects its viability and fertilization success [30,31]. Fertilization success in plants is results of a sequence of processes that takes place during the progamic phase [11]. Fertilization success and fruit set depends on many internal but also ecological factors including adaptation to changeable environmental conditions. On the other hand, according to Gillet and Hans Rolf [32], it is more important to produce ovules with viable egg cells than numerous pollen grains for plants.

Pollination, at the later flowering period can affect the reduction of fertilization effectiveness and thus, fruit setting [33]. Williams [29] indicated that viability of macrogametophyte (embryo sac) is a crucial component that affects the effective pollination period and fertilization process, allowing satisfactory yield in fruit species. Low ovule fertility in some pear cultivars could be a main reason for the poor yields, frequently connected with other factors. Pollen and ovule traits are also involved in assessing effective pollination period under diverse environmental conditions [29]. All of the above components and events could play an important role in year to year cropping variability. The choice of appropriate locations with favorable climatic conditions can greatly help in making flawless conditions for pollen transfer, pollen tube elongation, and consequently fruit [34]. 

For determination of the most efficient pollinizers for the pear cultivars ‘Ingeborg’ and ‘Celina’, the following methods have been applied so far: fluorescent analysis of progamic phase of fertilization [5,35], examination using microsatellite markers [6,36,37], and study of flowering performance and phenology [38]. So, as a follow up from the previous manuscript where pollen tube growth in different crossing combinations of ‘Ingeborg’ and ‘Celina’ was described [5], the aim of this work was to investigate the functionality of the embryo sacs, from developing, through viability, and up to the fertilization success and fruit set of the diploid (‘Celina’) and the triploid (‘Ingeborg’) pear cultivars, that can affect irregular yields of these cultivars. Completing knowledge of the reproductive biology of these two cultivars will give better possibility for the producers to get high and stable yields of good fruit quality in pear orchards under climate change. 

## 2. Results

### 2.1. Flowering Time and Climate Conditions 

**Figure 1:**
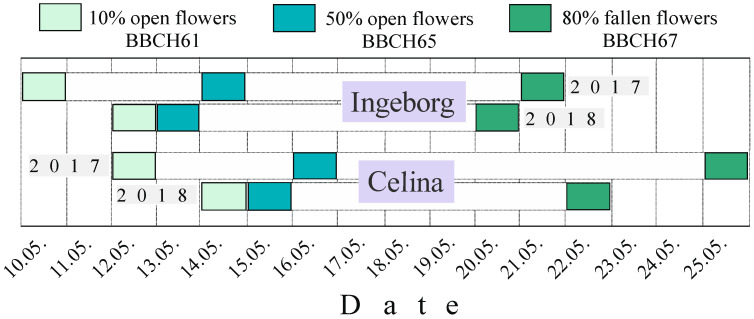
Flowering phenophases of pear cultivars ‘Ingeborg’ and ‘Celina’ in 2017 and 2018.

During 2017, the flowering of cultivar ‘Ingeborg’ lasted 12 days, having full bloom on 14 May, two days before ‘Celina’ (16 May) (Figure 1). 

In this year ‘Celina’ had 14-day flowering interval (12–25 May). In 2018 ‘Ingeborg’ flowered 9 days, showing full bloom on 13 May, two days before ‘Celina’ (15 May). The flowering period in cultivar ‘Celina’ was nine days (14–22 May). 

**Figure 2:**
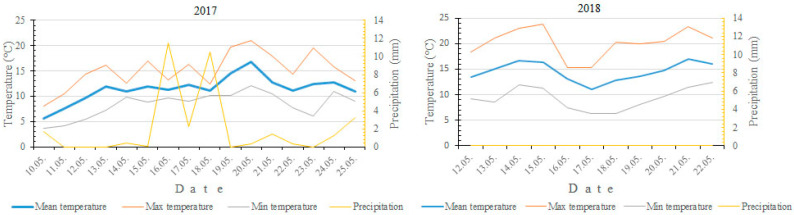
Temperatures and precipitation during flowering phenophases of pear cultivars ‘Ingeborg’ and ‘Celina’ in 2017 and 2018.

Mean temperatures during flowering period of both cultivars was 11.5 °C in 2017, while in 2018 it was 3.1 °C higher (14.6 °C) (Figure 2). During the whole flowering period, the average rainfall in 2017 was 2.1 mm/day, while in 2018 there was no rain in the same period. 

In 2017 the average monthly temperatures for March and April were 3.6 °C and 5.7 °C, respectively. For the same months the temperatures in 2018 were 0.3 °C and 6.3 °C higher. Warm and dry weather during flowering period that was in 2018 was completely unusual for western Norway. Moreover, higher post-bloom temperatures were recorded in 2018. The average temperature during the seven-day post-bloom period in 2017 was 13.1 °C (with a maximum daily temperature of 21.7 °C), whereas in 2018, it was 16.3 °C (with a maximum daily temperature of 29.4 °C). The precipitation was much higher in May of 2017 (51.0 mm) compared to 2018 (20.6 mm), but most of it was concentrated in the first 3–4 days of May, thus not disrupting the pollination of pear flowers.

### 2.2. Development of Embryo Sac

**Figure 3:**
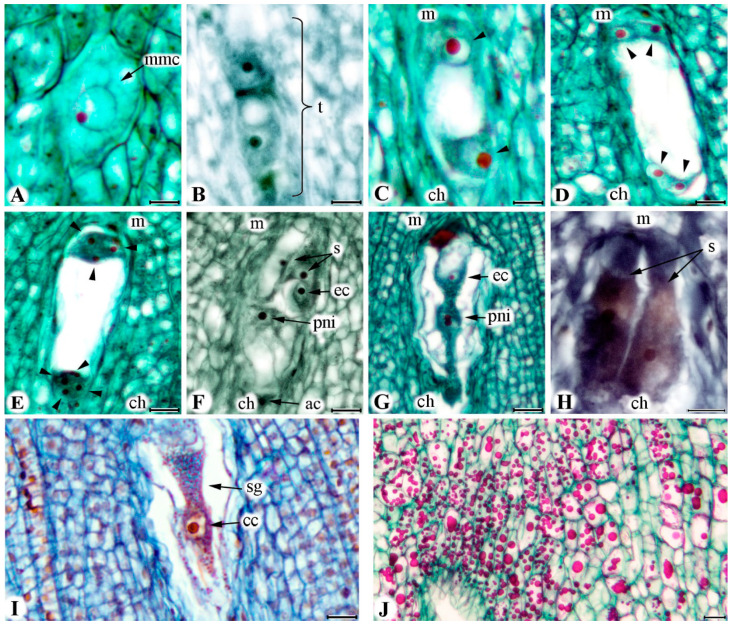
Development of embryo sac in pear cultivars ‘Ingeborg’ and ‘Celina’. (**A**) Megaspore mother cell—‘Ingeborg’. (**B**) Four megaspores in linear position—‘Celina’. (**C**) Two nuclei obtained after the first mitotic division of the functional megaspore—‘Ingeborg’. (**D**) Four-nuclei state after the second mitotic division—‘Ingeborg’. (**E**) Eight-nuclei state after third mitotic division—‘Ingeborg’. (**F**) Mature eight-nucleate embryo sac—‘Ingeborg’. (**G**) Normal position of egg cell and two polar nuclei—‘Celina’. (**H**) Elongation of synergids—‘Celina’. (**I**) Accumulation starch grains and nucleus of central cell—‘Ingeborg’. (**J**) Starch grains in tissue of ovary—‘Celina’. (mmc = megaspore mother cell; t = tetrad; m = micropylar pole; ch = chalazal pole; arrowheads = nuclei; s = synergid cells; ec = egg cell; pni = polar nuclei; ac = antipodal cell; sg = starch grains; cc = central cell nucleus). Scale bars = 0.01 mm.

All analysed pear ovaries in this study consisted of five locules, rarely more, with common axis from which ovules are attached. The ovules were characterized by anatropic position, bitegmic and crassinucellate type of integuments and short funiculus. Megaspore mother cells underwent the process of megasporogenesis where tetrads or megaspores were formed (Figure 3A,B). A linear order of megaspores was rarely observed at the beginning of flowering (BOF), indicating that these stages started earlier. During the process of megagametogenesis, megaspore enlarged, while nucleus went under three mitotic cycles (two, four, and eight nuclei), and formed embryo sac (BBCH stages 60–61) (Figure 3C–E). Mature embryo sac contained egg apparatus with two disposed side by side pear-shaped synergids with large vacuoles and nuclei in their upper parts (BBCH stage 65) (Figure 3F). The egg cell was located between synergids, having large vacuole and nucleus located toward the chalazal end of the embryo sac and with completely opposite polarity compared to synergids (Figure 3F,G). Central cell contained two polar nuclei (Figure 3F,G). Three ephemeral antipodal cells were situated at the chalazal pole of embryo sac (Figure 3F). 

**Figure 4:**
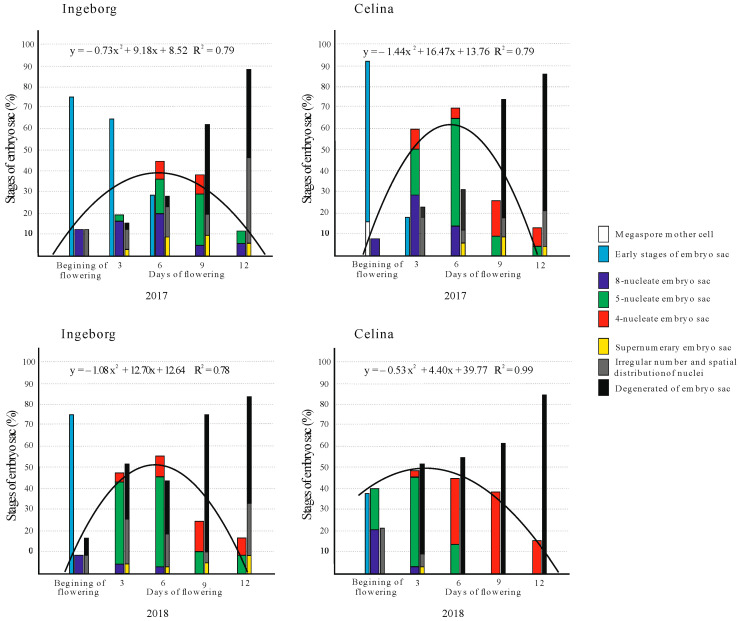
Regression analysis of regular embryo sac stages (%) and embryo sacs with different anomalies and degenerations (%) in unpollinated flowers at the BOF, and on the third, sixth, ninth and twelfth day after BOF in ‘Ingeborg‘ and ‘Celina‘ during 2017 and 2018.

At BOF, mature embryo sacs with eight nuclei were present in both cultivars and years. Highest number of these embryo sacs were recorded in 2017 (76%) compared to 2018 (38%), both in ‘Celina’ (Figure 4). In 2017, cultivar ‘Ingeborg‘ had 75% of eight nucleate embryo sacs on the BOF as well as 64.5% of those embryo sacs on the third day after BOF. The antipodes rapidly degenerated and became obscured between the third and sixth day after BOF (5-nucleate embryo sac), except for ‘Ingeborg’ where antipodes could be observed until the twelfth day after BOF in 2017. The fusing of polar nuclei into one central nucleus finally determined the number of nuclei in the embryo sac just before the process called syngamy (4-nucleate embryo sac). This process began on the third day after BOF (except for ‘Ingeborg’ in 2017, where it started on the sixth day after BOF). The average number of embryo sacs with four nuclei was the highest in cultivar ‘Celina’ (38.5%), on the ninth day after BOF in 2018 (Figure 4). 

**Figure 5:**
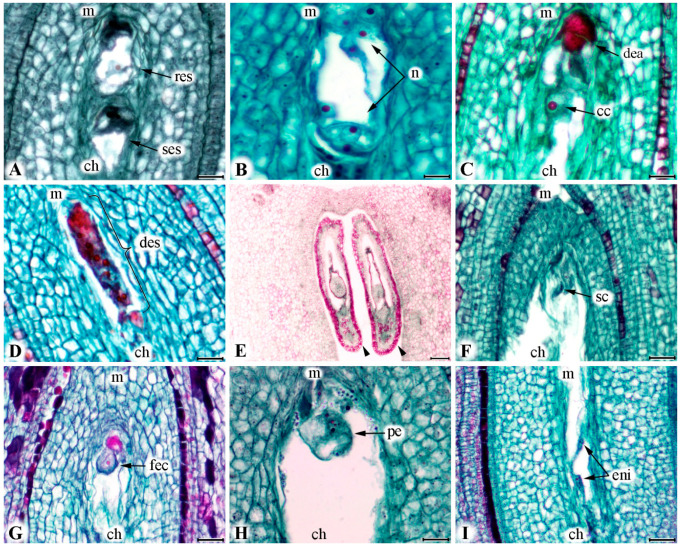
Development of embryo sac in pear cultivars ‘Ingeborg’ and ‘Celina’. (**A**) Regular embryo sac and supernumerary embryo sac—‘Ingeborg’. (**B**) Irregular number and spatial distribution of nuclei in embryo sac—‘Ingeborg’. (**C**) Degenerating of egg apparatus and central nuclei—‘Celina’. (**D**) Occurrence of degeneration of all elements of embryo sac—‘Ingeborg’. (**E**) Degeneration of ovules—‘Ingeborg’. (**F**) The sperm cells inside pollen tube—‘Celina’. (**G**) Fertilized egg cell—‘Ingeborg’. (**H**) Early embryogenesis—‘Celina’. (**I**) Endosperm nuclei—‘Ingeborg’. (m = micropylar pole; ch = chalazal pole; res = regular embryo sac; ses = supernumerary embryo sac; *n* = nuclei; dea = degenerated egg apparatus; cc = central nuclei; des = degenerated embryo sac; arrowheads = degenerated ovules; sc = sperm cells; fec = fertilized egg cell; pe = pro-embryo; eni = endosperm nuclei). Scale bars = 0.01 mm.

The typical elongation of the embryo sac and synergids began on the sixth day after BOF (Figure 3H). The accumulation of different size starch grains was noticed in the cytoplasm in and around nucleus of central cell and ovary tissue on the sixth day after BOF (Figure 3I,J). Also, in both cultivars, the appearance of supernumerary embryo sacs was detected, but was more pronounced in the triploid cultivar ‘Ingeborg’ (the highest value was 9.5% in 2017) (Figure 5A). Those kinds of additional embryo sacs most probably developed from supernumerary archesporium cells just below the primary embryo sac. Irregular shape and spatial position of embryo sac’s components occurred in high percentage in supernumerary embryo sacs. Embryo sacs with eight, five, and four nuclei that were regularly arranged represented successive functional stages in which the process of syngamy could finally take place.

### 2.3. Viability of Embryo Sac

Different signs of embryo sacs degeneration were observed during the entire flowering period. Degeneration, irregular number and spatial distribution of individual nuclei in egg cell were observed in both cultivars and years (Figure 5B). The degeneration of individual cells inside of the embryo sac was accompanied with the loss of their normal shape which was noticed on histological preparation as a strong colour reaction (Figure 5C). Occasionally, the whole egg sac showed a strong stain reaction which was a sign that it underwent complete degeneration (Figure 5D). Loss of viability of embryo sac was recorded immediately at the BOF in both cultivars and in both years. Degenerated ovules increased progressively after the sixth day after BOF and were very numerous at the twelfth day after BOF. The central nucleus showed the longest viability within embryo sacs, up to the twelfth day after BOF. Parallel to the above described degenerations of embryo sacs, the ovule tissue also showed signs of degeneration (Figure 5E) (in most cases at the tips of both outer and inner integument, and later spreading toward the whole ovule). 

All three functional stages of the embryo sac that are capable for fertilization (8-, 5- and 4-nucleate) are presented cumulatively with a regression line of the parabola type, which presents a trend of embryo sacs viability during flowering in a best way (Figure 4). The values of the multiple correlation coefficient R, ranking form 0.78 (nonpollinated ‘Ingeborg‘) to 0.99 (nonpollinated ‘Celina‘) in 2018, showed the studied stages were highly correlated. Regression lines were best fitted, and their trend showed maximum in ‘Ingeborg’ on the sixth day after BOF in 2017 and 2018 (44% and 56.3%, respectively). The highest average (68.9%) of embryo sac’s functional stages in cultivar ‘Celina‘ was recorded on the sixth day after BOF in 2017, while in 2018 it was on third day after BOF (48.3%). In this cultivar, the number of functional stages was the highest between the third and the sixth day after BOF in both years. 

In ‘Ingeborg’, as the flowering continued, the number of embryo sac’s functional stages decreased drastically and on the twelfth day after BOF it showed very low values of 11.8% (2017) and 16.6% (2018). The number of functional stages also decreased in ‘Celina’ and on the twelfth day after BOF it was 13% in 2017 and 15.4% in 2018. In 2018, in ‘Celina‘, the presence of 4-nucleate stage of embryo sacs (ready for process of syngamy) slowly decreased from sixth up to the twelfth day after BOF.

### 2.4. Fertilization Success 

It is common that synergids degenerate before the pollen tube arrives, but in some rarely cases pollen tube with male gametes can entered embryo sac which is situated between the two synergids without damaging it (Figure 5F). Fertilized egg cells were larger; more colored, as well as with more nucleoli (Figure 5G). The formation of early embryo and appearance of endosperm are main development stages in the process of early embryogenesis (Figure 5H). Endosperm formation usually overlapped with the process of embryo morphogenesis (Figure 5I). 

**Figure 6:**
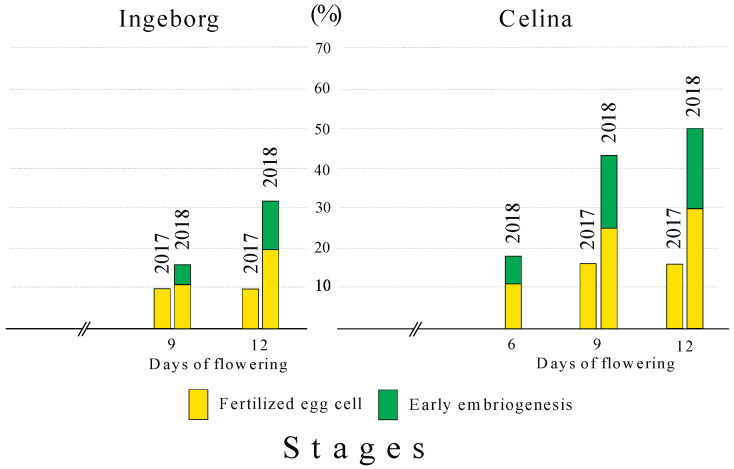
Percentage of embryo sacs with fertilized egg cell or with early embryogenesis in open-pollination of pear cultivars ‘Ingeborg’ and ‘Celina’ during 2017 and 2018. The standard deviation (SD) of studied traits presented in percentages was 2–3%.

Uneven fertilized egg cell and early embryogenesis were noticed in particular time of flowering, in both years of study and both cultivars. In 2017, ‘Ingeborg’ had only 9.1% of embryo sacs with fertilized egg cells that could be observed on the ninth and twelfth day after BOF (Figure 6). The following year, 20% embryo sacs had fertilized egg cell while another 12% embryo sac showed stages of early embryogenesis in the twelfth day after BOF. In 2017, the percentage of embryo sacs with a fertilized egg cell was higher in ‘Celina’ (16.7%) in relation to ‘Ingeborg’. The following year fertilized embryo sac and stages of early embryogenesis were already noticed in ‘Celina’ from the sixth day after BOF. In this cultivar, 50% of embryo sacs were either with fertilized egg cell or had a stage of early embryogenesis on the twelfth day after BOF (29.2% and 20.8%, respectively). 

### 2.5. Fruit Set 

**Figure 7:**
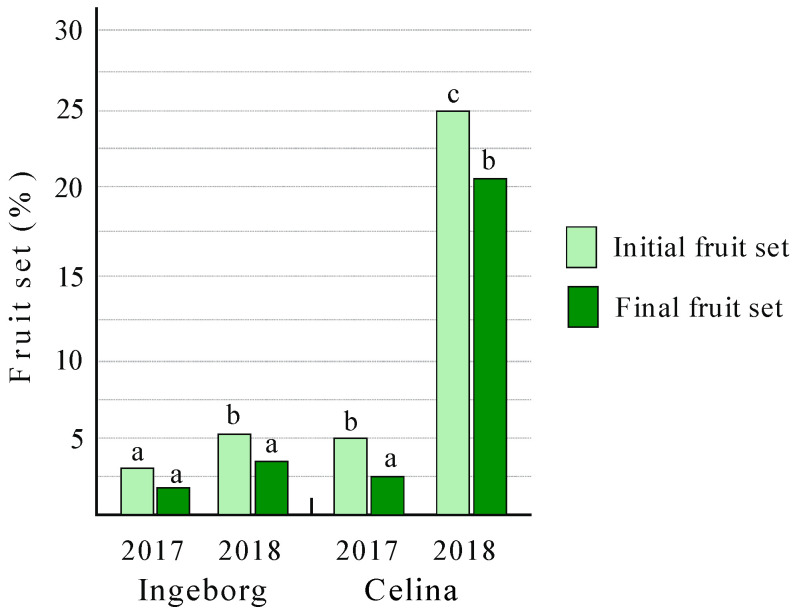
Fruit set in pear cultivars ‘Ingeborg’ and ‘Celina’ in open-pollination combination, during 2017 and 2018. Different letters above the bars denote a significant difference between the cultivars (for each trait studied) according to the LSD test, *p* < 0.05.

The values for initial and final set during open pollination are given in Figure 7. In 2017, values of final fruit set in both cultivars were low, for ‘Ingeborg’ (1.8%) and ‘Celina‘ (2.5%). In the following year, the difference in final fruit set between the cultivars was much more pronounced. In this second year, fruit set in ‘Celina‘ was 21.3%, while in ‘Ingeborg‘ it was more than six-fold lower (3.4%).

These results suggested that environmental conditions, especially temperature, may have influenced fruit set in the open-pollinated flowers.

## 3. Discussion

### 3.1. Developing and Viability of Embryo Sac 

Studies on the reproductive behavior of pear cultivars ‘Ingeborg‘ and ‘Celina‘ are most important in order to understand their adaptation to different environmental conditions, particularity in a specific context of Nordic climate. In addition to other factors, the efficiency of the progamic phase of fertilization, effective pollination period (EPP), success of fertilization, and embryo/seed development are necessary for successful set and high yields [39]. Pear ovules contained monosporic embryo sacs with eight nuclei, which are classified as polygonum type, and which is the most common type in seed plants [40]. Generally, the embryo sac is one of the most important factors in the reproductive process of angiosperm, because it conducts pollen tube guidance, gametes merging, and embryo/seed development [41]. In apple, the embryo sac development is associated with large morphological expansion of the flower [42]. 

In this study, in both cultivars and years of study, synergids first begin to degenerate one by one just after the sixth day after BOF, followed by degeneration of the egg cell. During this period, a large accumulation of starch grains can be seen in the central cell in addition to those already present in the ovary tissue. The aggregation and transfer of starch reserves to the nucellus and integuments show streaming of starch grains along, and outside the embryo sac [43]. Large amounts of starch grains during ovule maturation were directly related to their nutritive role in the process of fertilization and early embryogenesis [44,45]. 

Generally, the synergids are highly specialized and physiologically active cells involved in secretory, chemotropic, and transport of male gametes within the egg apparatus [46]. In this study the antipodes were ephemeral and are rarely presented after the sixth day after BOF. In many flowering plants, antipodal cells are those that have no clear function and their degeneration (programmed cell death) happens during embryo sac maturation, but before fertilization [47]. The polar nuclei may partially fuse with each other in the center of the nucellus before they are fertilized by a single sperm nucleus [43]. In this study the polar nuclei and central nucleus showed the longest viability, even when surrounding tissue of embryo sac showed complete degeneration (both integuments and nucellus of ovule). 

During the process of embryo sac maturing, some usual developmental structures of 8-, 5-, and 4-nucleus functional stages were noticed between the third and sixth day after BOF, which were cultivar- and year-dependent. The highest average value (68.9%) of the most functional embryo sac stages was recorded in cultivar ‘Celina‘, on the sixth day after BOF in 2017. Also, this cultivar showed the longest interval of the persistence of 4-nuclei stage of embryo sacs, which lasted from the sixth until the twelfth day after BOF in 2018. 

Loss of viability of embryo sac was recorded immediately at the beginning of full flowering in both cultivars and in both years. Degenerated ovules increased progressively after the sixth day after BOF and were numerous on the twelfth day after BOF. Similarly, Stösser and Anvari [48] recorded different viabilty of ovules between third and the fifth day of flowering. Degeneration of the embryo sac nucleuses and irregular spatial distribution of egg cell and synergids, mostly separation, was noticed. According to [30,31] this is tipical for some other fruit species, like sour cherry and plum. Two-year average of functional stages of embryo sac (8-, 5- and 4- nucleate) during full flowering was just 28% in ‘Ingeborg‘ and 36.2% in ‘Celina‘. In contrast to pear, in stone fruits, viable embryo sacs can disappear up to sixth day after BOF [49], persist up to twelfth day after BOF [31,50], or the development of the embryo sacs can be slowed down so that a high percentage of ovules without differentiated embryo sacs can be found [51]. This means that a certain degree of megagametophyte development at full bloom may not be enough to ensure a good crop, since high percentages of functional ovules can have a low fruit set [52]. So, high yields require much more gamete production resources in ovules compared to pollen. 

Type of pollination (controlled or open) also affected the potential of viability of embryo sac. Delayed differentiation of the megaspore, a lack of polar nuclei fusion, and their abortion can be seen in self and open-pollination plant species [53]. The functional female gametophyte, which is genotype dependent, plays a critical role in essentially every step of the reproductive process, directly affecting successful fertilization and fruit set [14,54]. In this study, the phenomenon of supernumerary abnormal embryo sac was noticed in to 9.5% ovules and was more pronounced in the triploid cultivar ‘Ingeborg’ in 2017. These embryo sacs did not interfere with the development of the primary embryo sacs, but it remained unknown if embryos were formed in primary or secondary embryo sac, or if it possible to have embryos in any of those two embryo sacs at all. Generally, it is known that triploids are infertile, due to the three sets of chromosomes and their unbalanced segregation during meiosis. Very often, seeds are just having apomictic embryos [28,36].

### 3.2. Fertilization Success and Fruit Set 

A process from pollination, via pollen elongation to fecundation varies between fruit species [30]. It means that male and female gametes must be in the same phase of the cell cycle, in order to have successful mating [55]. The appearance of early embryo and endosperm formation in this study was climatic conditions dependant. In 2017 it was on the ninth day after BOF while in 2018 it was the sixth day after BOF. The earliest occurrence of fertilized ovules and early embryogenesis was recorded in ‘Celina‘, on the sixth day after BOF in 2018. In ‘Celina’ the number of fertilized ovule and early embryogenesis was higher in 2018 compared to 2017. These results can be explained by the fact that the average daily temperature during full bloom in 2018 was 14.6 °C, which was 3.1 °C higher than in 2017 (11.5 °C). However, meso- or micro-climatic conditions could have some effect too. Temperature has been pointed out as the main factor interacting with the whole or just part of a reproductive process [8,54]. This was previously proved in a study of Cerović et al. [5] who tried to find the best pollinizers for ‘Ingeborg’ and ‘Celina’ under the same environmental conditions and concluded that all crossing combinations were more pronounced in cultivar ‘Celina’ in 2018 due to much warmer weather. Besides the temperature, the higher number of 5-nucleate and 4-nucleate stage of embryo sacs also contributed to the higher fertilization success of ‘Celina’ in 2018 (Figure 6). 

The pollinator activity, climatic conditions, efficient and compatible pollinizers, adequate pollinizers, viability of ovule/egg cell, and fertilization success are being crucial factors for the fruit set. Higher number of initial and final fruit set was obtained in ‘Celina‘ (25% and 21.3%, respectively) in 2018, which was five and eight fold, respectively, higher than in 2017. This is already proved by Nyéki [56] who noticed great differences among the pear cultivars regarding fruit set in open-pollinated flowers. 

In our study, values of fertilization success had direct influence on fruit set. Also, values of fertilization success and fruit set showed the same relative relationship in both cultivars and years of study. Low fruit set in ‘Ingeborg’ was expected, since this cultivar is triploid, and these kinds of genotypes sometimes show no measurable female fertility [28]. Irregular chromosome distribution in triploids can lead to unbalanced genetic composition associated with different levels of aneuploidy of embryo sac elements. This indicates the occurrence of zygote and embryo abortion.

The functionality and viability of the embryo sacs have been identified as the limiting factors of EPP in apples and pears [29,57]. If climatic conditions are unfavorable, pollination can be delayed, and then fertilization process lean on ovules that stayed viable. Thus, it is very important to know the climate conditions, in particular temperatures, because an optimal temperature regime is necessary for high fertilization success and fruit set. In our study, the results of fruit set after open pollination showed the genetically determined features of those cultivars. 

## 4. Materials and Methods 

### 4.1. Plant Material and Experimental Design

The pear cultivars ‘Ingeborg’ (‘Conference‘ × ‘Bonne Luise‘) and ‘Celina‘ (‘Colorée de Juillet‘ × ‘Williams’) were used for the study of embryo sac functionality in relation to fertilization success and fruit set. Investigation was done at the Njøs Fruit and Berry Center, Leikanger, Western Norway at latitude 61°10′43.2″ N, longitude 6°51′34.3″ E, during two consecutive years (2017–2018). Orchard planting and maintenance has already been described in Cerovic et al. [5].

For this experiment ten trees per each cultivar were used. On each tree 24 flowers were emasculated (6 inflorescences with four flowers) where the whole periant was removed (balloon phase, BBCH 59) and left unpollinated for analysis of embryo sac functionality (development and viability), and another 24 flowers were counted and tagged, and left for open pollination in order to study fertilization success. In total, 240 flowers were used for embryological studies per cultivar. After emasculations, flowers were kept unbagged, in order to avoid damage of pistil/ovule especially in rainy or windy days. Randomly selected, both long and spur branches were used, with normally formed inflorescences. For each sampling date, one flower/ovule per inflorescence was picked (15 × king flower/ovule, 15 × first lateral flower/ovule, 15 × second lateral flower/ovule and 15 × third lateral flower/ovule). During the experiment, bumble bees, wild bees, black bees, wasps, and hoverflies were present in the orchard as pollinators.

### 4.2. Flowering Time and Climate Conditions 

Flowering phenophases, which started in the first half of the May, were followed as previously described by Cerovic et al. [5] according to the BBCH scale [58]. Climatic data were gathered from the meteorological stations located in Njøs which was 200 m distant from pear orchard.

Western Norway is characterized by cool summers and mild winters, having average temperature of 6.6 °C, with 994 mm rainfall. Weather fronts are usually coming from the Atlantic Ocean, so that clouds, rain, and wind dominate throughout the year. Regardless of whether the Gulf Stream is moderating its climate, cold temperatures can occur during spring.

### 4.3. Microscopic Preparations

Flowers were picked from BOF, and on the third, sixth, ninth and twelfth day after the beginning of this phenophase. The flowers/ovaries were fixed and embedded according to Cerović and Mićić [30]. Triplet staining with Safranin, Cristal light, and Light Green SF Yellowish according to Gerlach [59] was used in this study. Safranin coloured lignified cell walls, cell nucleolus and starch grains with red, while light green SF coloured cytoplasm and cellulose cell walls in different shades of green. The crystal violet differentiated safranin color. Histological preparations were observed under the light microscope Optic 900T with TCA1000-C color camera (Colo, Slovenia). 

### 4.4. Fruit Set

A group of 250–300 open-pollinated flowers in both cultivars were chosen to test fruit set. The percentage of initial fruit set was counted around one month after full bloom (before the ‘June’ drop), while final fruit set was recorded just before the harvest (BBCH 79). Harvesting time of cultivar ‘Ingeborg’ was on September 25 in 2017 and August 30 in 2018. Cultivar ‘Celina’ was harvested on September 15 in 2017 and August 20 in 2018. The initial/final fruit set was calculated as a percentage of formed/harvested fruits per 100 flowers.

### 4.5. Statistical Analysis

Two factorial analyses of variance (ANOVA) were used to process the obtained data. Differences were compared using LSD test (LSD 0.05 = 95% confidence). The application of regression allowed determining the best trend between functional stages of embryo sacs i.e., their viability and days of flowering. Statistical analyses were performed using the STATISTICA for Windows 6.0 (StatSoft Inc., Tulsa, Okla, USA) software package.

## 5. Conclusions

The functionality of embryo sac-developing, viability, and fertilization success in relation to fruit set was examined for two years in diploid (‘Celina‘) and triploid (‘Ingeborg‘) pear cultivars under the climate conditions of western Norway, as a follow up to previous studies. 

In order to improve the efficacy of pollinizers, it was necessary to improve the knowledge of the impact and role of female factors on reproductive biology of these two cultivars. The flowering and fruit set of these two cultivars grown under identical agro-environmental conditions was cultivar and genetic plasticity dependent, and its adaptability to environmental conditions dominated particularly in years with greater variation in temperature conditions.

Our study showed low variability of qualitative and quantitative traits in developing embryo sacs and their viability in both cultivars during the first twelve days after BOF. Unlike previous parameters, values for fertilization success and fruit set were higher in the second year of study due to the higher average temperature during flowering. The difference regarding fertilization success and fruit set was more pronounced in cultivar ‘Celina‘. This characteristic probably indicates the existence of better adaptation in relation to high temperatures in this cultivar. In contrast, cultivar ‘Ingeborg’ has a genetic load associated with low temperature adaptation, which indicated that the functioning of the embryo sac is cultivar dependent. This confirmed the presence of synchronized processes of micro- and macro-gametes development and gametes fusion, which influenced a higher fruit set and yield in pear cultivar ‘Celina‘. 

Unfortunately, since we studied just one diploid and one triploid pear cultivar, we couldn’t prove if the differences between ‘Celina’ and ‘Ingeborg’ are also connected with the chromosome number. To better distinguish between diploids and triploids, much more studies using additional diploid and triploid pear cultivars should be done.
